# Xenohormone transactivities are inversely associated to serum POPs in Inuit

**DOI:** 10.1186/1476-069X-7-38

**Published:** 2008-07-15

**Authors:** Tanja Krüger, Mandana Ghisari, Philip S Hjelmborg, Bente Deutch, Eva C Bonefeld-Jorgensen

**Affiliations:** 1 Unit of Cellular and Molecular Toxicology, Centre for Arctic Environmental Medicine, Institute of Public Health, University of Aarhus, Vennelyst Boulevard 6, Build 1260, 8000 Aarhus C, Denmark

## Abstract

**Background:**

The persistent organic pollutants (POPs) are highly lipophilic and resistant to biodegradation and found in e.g. seafood and marine mammals. Greenlandic Inuit have high intake of marine food and thus high POP burden that varies according to local conditions and dietary preference. We do for the very first time report the serum POP related non-steroidal xenohormone activity of Inuit across Greenland.

**The aims **were 1) to determine the integrated xenohormone bioactivities as an exposure biomarker of the actual lipophilic serum POP mixture measuring the effect on estrogen (ER) and androgen receptor (AR) transactivity in citizens from different Greenlandic districts and 2) to evaluate associations to serum POP markers (14 PCBs and 10 pesticides) and lifestyle characteristics.

**Methods:**

Serum samples from 121 men and 119 women from Nuuk, Sisimiut and Qaanaaq were extracted using SPE-HPLC fractionation to obtain the serum POP fraction free of endogenous hormones. The serum POP fraction was used for determination of xenohormone transactivity using ER and AR reporter gene assays.

**Results:**

In overall, the xenohormone transactivities differed between districts as well as between the genders. Associations between the transactivities and age, n-3/n-6 and smoker years were observed. The xenoestrogenic and xenoandrogenic transactivities correlated negatively to the POPs for the combined female and male data, respectively.

**Conclusion:**

The non-steroidal xenohormone transactivities can be used as an integrated biomarker of POP exposure and lifestyle characteristics. The actual serum POP mixtures antagonized the age adjusted sex hormone receptor functions. Comparison of different study populations requires in addition to age inclusion of diet and lifestyle factors.

## 1. Introduction

The increasing load of the environment by man-made pollutants is of concern for the human health. Due to long-range transport by atmospheric and oceanic currents [1,2] the human exposure is not limited to individuals living close to the sources of the contaminants. The lipophilic persistent organic pollutants (POPs) includes polychlorinated dibenzo-*p*-dioxins/furans (PCDDs/PCDFs), polychlorinated biphenyls (PCBs) and certain organochlor pesticide residues e.g. 2,2-bis(*p*-chlorophenyl)-1,1,1-trichloroethane (DDT), its metabolite 1,1-dichloro-2,2-bis (*p*-chlorophenyl)-ethylene (*p,p'*-DDE), toxaphenes and chlordanes. Due to their high lipophilicity and resistance to biodegradation POPs are biomagnified through the food chain and found in fatty tissues at high concentrations in predator fish and birds, seals, whales and polar bears [3]. Greenlandic Inuit display high body burden of POPs [4], that significantly correlate with age, smoking and the level of n-3 polyunsaturated fatty acids in plasma, being strong indicators of the main source of POP contaminants in their traditional marine food [5-9].

In some Greenlandic districts the PCB body burden levels exceed Health Canada guidelines of concern (5 – 100 μl/L plasma) [10]. Also within Greenland strong regional differences are observed with the highest contaminant levels found in Inuit at the East coast [10], where also higher levels of PCBs and DDT are found in marine species and birds compared to the West coast [11,12]. Some districts and settlements such as Qaanaaq (North West) still primarily rely on traditional foods, whereas the diet in the cities Nuuk and Sisimiut (South West) are more westernized [10,13].

During the last two decades, a number of POPs have been identified and characterized as being estrogenic, antiestrogenic and/or antiandrogenic [14-18], and to have a multitude of potential health effects on wildlife and humans, including immunotoxicity, carcinogenicity, and adverse effects on reproductive, neurobehavioral, and endocrine functions [19-24]. Recently also endocrine-related human health effects of POPs on child development were reported at the individual or population level [25-27]. However, the toxicological assessment of POPs on humans is complicated. Not only is the analytical chemical approach for the detection of all xenobiotics practically impossible especially on large-scale surveys but additive enhancement of hormone actions has been reported *in vitro *for xenoestrogen mixtures [28,29] and recently *in vivo *for antiandrogens [30]. Therefore, the assessment of the integrated biological effect of the actual chemical mixture in human blood is important and *ex vivo *cell systems have recently been introduced to enable the assessment of the integrated level of xenobiotic transactivity in human adipose tissue [20,31,32] or in human serum [33-37]. We have previously validated the SPE-HPLC serum POP extraction method and it was demonstrated that the ER- and AR-CALUX assays respond to a wide range of different compounds including PCBs and pesticides [14,15] as well as to PCB-spiked serum extracts [35].

**The aims **were 1) to determine the integrated xenohormone bioactivities as an exposure marker of the actual lipophilic serum POP mixture measuring the effect on estrogen (ER) and androgen receptor (AR) transactivity in cerum of citizens from different Greenlandic districts and 2) to evaluate associations of the xenohormone transactivities to serum POP markers (14 PCBs and 10 pesticides) and lifestyle characteristics.

## 2. Methods

### 2.1 Study population and collection of blood samples

The subjects and sampling methods have been described in detail elsewhere [7]. The study was designed to include 100 serum samples (50 men and 50 women) from each of the three districts Nuuk, Sisimiut and Qaanaaq. However, in some cases insufficient blood was collected for the analyses and some samples were lost in the SPE-HPLC extraction, resulting in a reduced number of serum samples from each district. All participants from Nuuk and Sisimiut in South West Greenland and Qaanaaq in North West Greenland were of Inuit decent, defined as having more than two grandparents born in Greenland. The data of the Sisimiut men was also a part of the EU project INUEDO. All participants completed a standard questionnaire including questions about demographic and lifestyle parameters. Venous blood samples were taken and prepared for determination of POPs and fatty acid profiles and stored at -80°C until analyzed as described [7].

### 2.2 Determination of POPs and fatty acids

Plasma samples were analyzed for POPs including cis-, trans- and oxy-chlordane, *p,p'*-DDE, *p,p'*-DDT, hexachlorobenzene (HCB), β-hexachlorocyclohexane (β-HCH), mirex, toxaphene 26, toxaphene 50 and 14 PCB congeners (CB28, CB52, CB99, CB101, CB105, CB118, CB128, CB138, CB153, CB156, CB170, CB180, CB183, CB187) by gas chromatography (GC) at the certified laboratory, Le Centre de Toxichologie, Sainte Foy, Quebec, Canada [38,7]. All determined POPs were adjusted to the plasma lipid content analyzed from the corresponding samples [38,7] and reported as μg/kg lipid.

The fatty acid profiles were determined in plasma phospholipids at the Biology Department, University of Guelph, Canada [9]. The n-3 polyunsaturated fatty acids were reported on the sum of C18:3, n-3, C20:4, n-3, C20:5, n-3, C22:5, n-3 and C22:6, n-3, and the n-6 fatty acids was the sum of C18:2, n-6, C18:3, n-6, C20:2, n-6, C20:3, n-6 and C20:4, n-6.

### 2.3 SPE-HPLC fractionation of the serum samples

To obtain the serum fraction containing the actual mixture of bio-accumulated lipophilic POPs a solid phase extraction (SPE) and high performance liquid chromatography (HPLC) fractionation was performed on 3.6 ml serum [35]. The first fraction (F1: 0.00 – 5.30 min.) was defined to include most POPs and free of endogenous hormones [35]. This SPE-HPLC F1 extract was evaporated and stored at -80°C for later ER or AR mediated CALUX assays.

A set of serum control samples were prepared by combining batches of male and female serum (blood bank of Aarhus Sygehus, DK), respectively, and distributed into 3.6 ml portions and stored at -80°C. One serum sample from each sex was, on a weekly basis, processed by the SPE-HPLC method in parallel with the study samples serving as serum control for the cleanup procedure.

On the day of analysis the SPE-HPLC F1 extracts (samples and controls) were thawed and processed as previously described [33,36]. The samples were analyzed randomly attempting to analyze samples from different districts in each independent assay.

### 2.4 Receptor chemically activated luciferase gene expression (CALUX) assays

SPE-HPLC serum extracts were analyzed in order to assess the ability of the lipophilic POPs to affect the ER or AR transactivity (referred to as XER and XAR, respectively). Moreover, we determined the effect of the serum extracts in the presence of the respective high affinity ligand of the receptors (the ER agonist 17β-estradiol (E2); the synthetic AR agonist methyltrienolone (R1881)) to mimic the effect *in vivo *assessing the ability of the lipophilic POPs to compete with the endogenous hormones/ligands (called XERcomp and XARcomp).

The ER transactivation response (ER-CALUX) were determined in the stable transfected MVLN cells, a derivative of the ER positive MCF-7 cell line, carrying the ERE-luciferase reporter vector as described previously [39,33]. AR-CALUX transactivity was determined in the Chinese Hamster Ovary cells (CHO-K1) by transient co-transfection with the mouse mammary tumor virus-luciferase (MMTV-LUC) reporter vector (kindly provided by Dr. Ronald M. Evans, Howard Hughes Medical Institute, San Diego, CA, USA) and the AR expression plasmid pSVAR0 (kindly provided by Dr. A.O. Brinkmann, Erasmus University, Rotterdam, The Netherlands) [36]. The determined luciferase activity per well was corrected for cell density using protein measurements and expressed in relative luciferase units per microgram protein (RLU/μg protein) equal to a response of 0.32 ml serum. The controls and the SPE-HPLC F1 serum extracts were determined at least in triplicate. If one of the triplicate values deviated more than 30% from the other two values, the mean was calculated from two wells only. The mean of the samples were related to the respective solvent controls.

In each independent assay a concentration-response control for the receptor ligand (E2 or R1881) was analyzed in parallel. The solvent controls ± E2 or R1881 were handled and analyzed in the same way as the SPE-HPLC F1 extracts [33,36]. The average ER-CALUX intra-coefficient of variation (CV) of the serum extracts and inter-CV of solvent controls were below 5%, and for AR-CALUX the intra-CV of the serum extracts and inter-CV of solvent controls were 11% and 14%, respectively. No cell toxicity was measured upon exposure of cells to SPE-HPLC extracts determined by CellTiter 96 Cell Proliferation assay from Promega (Madison WI, US) [33,36].

### 2.5 Statistical analysis

In each independent CALUX-assay transactivity differences between the triple serum extract determinations and their respective solvent controls (% agonistic, % decreased, % further increased and % antagonistic) were tested by the Student's T-test in Microsoft Excel (p ≤ 0.05).

To improve normal distribution the serum ER and AR transactivity and the POP data were natural logarithmic transformed for the statistical analysis. The analyses were performed on continuous data.

The comparisons of means between men and women were performed by Student's t-test. One-way ANOVA was used to compare levels of xenohormone transactivities and POPs among the districts. If differences were observed, multiple comparison *ad hoc *test were performed using the least significant difference (LSD) pair wise multiple comparison test for the variables with equal variance (p > 0.05) and Dunnett's T3 test for the variables with an unequal variance (p ≤ 0.05). The homogeneity of variance was tested by Levene's test.

Pearson's correlation analyses (two-tailed) were used to evaluate 1) the interrelationship between the 14 PCB congeners and the 10 pesticides and 2) associations between xenohormone transactivity and lifestyle characteristics.

Homogeneity or heterogeneity between the POP markers and xenohormone transactivity for each gender across the study groups were evaluated using multiple linear regressions analysis. Both the combined male or female data across the districts were found to be homogenous except for the XER activity of the combined men (see Additional file 1). Therefore, to obtain a better statistical power and the overall trend of the studied groups, the data based on the combined genders across the districts were evaluated assuming a common intercept.

To our knowledge no experience about which dietary and/or other life-style characteristics that might influence the xenohormone transactivity is reported. We hypothesized that a potential predictor of POP bioaccumulation might also be a potential predictor for serum xenohormone transactivity. As known from literature age and seafood intake affect the serum POP levels [40]. Also body mass index (BMI), smoking years and bird intake might influence the serum POP levels [7]. Using the multivariate linear regression model, assessing the relation between the xenohormone transactivities and the POPs the impact of the lifestyle characteristics were evaluated by entering variables together with the POPs. Due to the small sample sizes and to obtain a higher power this was done for the combined genders across the districts.

The statistical analysis was performed in SPSS 13.0 (SPSS Inc, Chicago, IL). The term statistically significant level is used to denote a p-value ≤ 0.05.

## 3. Results

### 3.1 Lifestyle characteristics of the study groups

Lifestyle characteristics that potentially influence the POP related ER and AR mediated transactivities of the volunteers are shown in Table 1. The age range of the volunteers was 18–72 years. The Nuuk men were older than the rest of the study groups, having similar age range. Furthermore, Nuuk men had the highest smoking year value, the highest n-3/n-6 fatty acid ratio and the highest level of seabird intake. BMI did not differ between districts or gender. Generally male participants had lower dairy food intake than women. Age and n-3/n-6 ratio was significantly correlated (p < 0.05) except for Nuuk women.

**Table 1:** Lifestyle characteristics

		**Men**				**Women**			
		
		Nuuk	Sisimiut	Qaanaaq	All	Nuuk	Sisimiut	Qaanaaq	All
Age (years)	n	32	51	38	121	45	42	32	119
	median	55	30	34	36	38	32	34	35
	mean	54	31	33	38	36	33	32	34
	min	38	18	19	18	19	18	18	18
	max	72	46	45	72	45	44	44	45
	P-value*	<.001	.15	.68	.001				
	P-value**	<.001 (Nuuk higher)				.07 (no post hoc test)			

BMI (kg/m^2^)	n	30	51	38	119	43	42	32	117
	median	28	26	27	27	26	26	24	25
	mean	27	27	27	27	26	27	25	26
	min	22	19	20	19	18	19	18	18
	max	35	36	41	41	32	47	34	47
	P-value*	.03	.92	.06	.04				
	P-value**	.40 (no post hoc test)				.56 (no post hoc test)			

Smoking (years)	n	29	51	37	117	42	42	30	114
	median	35	11	18	18	20	12	16	15
	mean	31	12	17	18	16	12	15	14
	min	0	0	0	0	0	0	0	0
	max	59	26	30	59	33	27	29	33
	P-value*	<.001	.85	.71	.001				
	P-value**	<.001 (all different)				.22 (no post hoc test)			

n-3/n-6	n	32	37	38	107	45	38	32	115
	median	0.55	0.22	0.39	0.35	0.25	0.29	0.37	0.29
	mean	0.69	0.29	0.50	0.48	0.29	0.32	0.43	0.34
	min	0.20	0.10	0.09	0.09	0.12	0.14	0.15	0.12
	max	1.65	0.73	1.45	1.65	0.72	0.82	1.26	1.26
	P-value*	<.001	.18	.95	<.001				
	P-value**	<.001(all different)				<.001 (Qaanaaq higher)			

Seabird intake (per month)	n	30	48	36	114	44	41	31	116
	median	2.0	1.0	1.0	2.0	1.0	1.0	1.0	1.0
	mean	4.6	2.7	2.4	3.1	1.4	1.3	3.5	1.9
	min	0	0	0	0	0	0	1	0
	max	20	28	20	28	8	2	28	28
	P-value*	<.001	.15	.84	<.001				
	P-value**	<.001 (Nuuk higher)				.02 (***)			

Dairy food consumption (per month)	n	31	50	38	119	45	42	32	119
	median	36	51	32	40	56	57	37	50
	mean	37	49	31	40	53	49	35	47
	min	4	8	3	3	2	0	0	0
	max	76	84	58	84	84	84	84	84
	P-value*	.005	.64	.34	.05				
	P-value**	<.001 (Sisimiut higher)				.01 (Qaanaaq lower)			

*: p value for men compared to women within the same district (e.g. Nuuk men and Nuuk women) using Student's t-test.

**: p value for comparison between the districts for the separate genders (e.g. Nuuk vs. Sisimiut vs Qaanaaq and vice versa) using ANOVA, result in the brackets (post hoc LSD/Dunnett T3 test).

***: Qaanaaq borderline higher than Sisimiut (p = 0.07)

### 3.2 POP levels and intercorrelations

For PCB28, PCB52 and PCB128 more than 68% of the samples were at the detection limit (0.01 μg), whereas 37% and 26% of the samples were at the detection limit for PCB101 and PCB105, respectively. 6% of the samples were at the detection limit for PCB156 and PCB183, while less than 1% of the remaining PCBs were at the detection limit.

High intercorrelations between the PCBs (r > 0.66) were observed except for PCBs of lower concentration: PCB28 (0.24 < r < 0.64), PCB52 (0.24 < r < 0.64) and PCB128 (0.54 < r < 0.74). The pesticides were highly intercorrelated (r > 0.75). However, different POP intercorrelations were observed for the study groups. Generally in Nuuk and Qaanaaq the intercorrelations among PCBs and pesticides were higher than in Sisimiut, and higher intercorrelations were observed for men compared to women.

Due to difference in chemical structure and toxicological properties we grouped the POPs as following: ΣPCB-12 (all PCBs except PCB28 and PCB52), ΣPCB-estrogenic (PCB28 + PCB52 + PCB99 + PCB 101) [41], ΣPCB-antiestrogenic (all PCBs except PCB28 + PCB52 + PCB99 + PCB 101), ΣDL-PCB (dioxin like PCB105 + PCB118 + PCB156), ΣPCB, Σchlordanes, Σtoxaphenes, DDT+DDE, Σpesticide and total ΣPOP. However, since the results regarding the associations between xenohormone transactivities and the defined POP groups were similar, we only show the results for ΣPCB, Σpesticide and ΣPOP.

Male participants had significantly higher POP levels than women for all districts (Table 2). For the men, similar median levels of ΣPCB, Σpesticide and ΣPOP were observed in Nuuk and Qaanaaq, being significantly higher than in Sisimiut. Qaanaaq women had significantly higher POP levels than that of Nuuk and Sisimiut women as also reported by Deutch et al [7].

**Table 2:** Lipid adjusted serum concentrations of the POPs

		**Men**				**Women**			
		
		Nuuk	Sisimiut	Qaanaaq	All	Nuuk	Sisimiut	Qaanaaq	All
	n	32	51	36	119	44	42	30	116
ΣPCB (μg/kg lipid)	median	2,680	540	1,830	1,350	300	350	1,190	450
	mean	3,040	750	2,630	1,940	490	460	1,250	680
	min	490	130	620	130	97	67	99	67
	max	11,100	2,370	7,100	11,100	2,430	1,380	3,490	3,490
	P-value*	<.001	.001	<.001	<.001				
	P-value**	<.001 (Sisimiut lower)				<.001 (Qaanaaq higher)			

	n	32	51	36	119	44	42	30	116
Σpesticide (μg/kg lipid)	median	3,500	740	2,590	1,590	480	500	1,670	640
	mean	4,070	1,010	3,740	2,660	680	700	1,890	1,000
	min	410	140	620	140	84	76	78	76
	max	19,100	3,540	14,400	19,100	2,880	3,010	7,530	7,530
	P-value*	<.001	.01	.002	<.001				
	P-value**	<.001 (Sisimiut lower)				<.001 (Qaanaaq higher)			

	n	32	51	36	119	44	42	30	116
ΣPOP (μg/kg lipid)	median	6,420	1,360	4,390	3,020	760	830	2,850	1,110
	mean	7,250	1,770	6,380	4,640	1,190	1,170	3,140	1,690
	min	910	290	1,240	290	180	150	190	150
	max	30,800	5,910	19,900	30,800	5,370	4,350	10,800	10,800
	P-value*	<.001	.003	<.001	<.001				
	P-value**	<.001 (Sisimiut lower)				<.001 (Qaanaaq higher)			

ΣPCB: sum of 14 PCB congeners. Σpesticide: sum of the 10 pesticide residues. ΣPOP: sum of all 24 POPs.

*: p value for men compared to women within the same district (e.g. Nuuk men and Nuuk women) using Student's t-test.

**: p value for comparison between the districts for the separate genders (e.g. Nuuk vs Sisimiut vs Qaanaaq and vice versa) using ANOVA, result in the brackets (post hoc LSD/Dunnett T3 test).

Because of the differences between the genders and to improve the statistical power we mainly focus on the combined male and combined female data across districts.

### 3.3 Xenoestrogenic transactivities

In overall the serum POP extracts of both sexes predominantly decreased/antagonized the ER (XER) and E2-ER induced transactivities (XERcomp), respectively, and for all districts except Nuuk women (having equal % of further increased and antagonized samples) the median XERcomp value was below the E2 reference control level (Table 3). No differences in the xenoestrogenic activities were observed between men and women except for the XERcomp activity of Sisimiut men being significantly lower than Sisimiut women (Table 3). The ANOVA test showed in overall significant differences between the districts for both sexes (Table 3).

**Table 3:** Xenoestrogenic and xenoandrogenic transactivities of the serum POP fraction

		**Male serum**				**Female serum**			
		
		Nuuk	Sisimiut	Qaanaaq	All	Nuuk	Sisimiut	Qaanaaq	All
**XER **RLU/μg protein	*n*	32	51	36	119	45	42	32	118
	median	1.00	0.95	0.97	0.97	1.05	0.90	1.01	0.99
	mean ± SD	1.00 ± *0.08*	0.94 ± *0.08*	0.98 ± *0.09*	0.97 ± *0.09*	1.03 ± *0.08*	0.94 ± *0.23*	1.00 ± *0.10*	0.99 ± *0.16*
	% agonistic	6	2	14	7	4	5	6	5
	% decreased	9	33	25	24	9	20	28	18
	P-value*	.09	.59	.24	.07				
	P-value**	.02 (Nuuk and Sisimiut different)				.001 (Sisimiut different)			

**XERcomp **RLU/μg protein	*n*	32	51	38	121	45	42	32	118
	median	0.97	0.82	0.93	0.89	1.03	0.96	0.91	0.96
	mean ± SD	0.99 ± *0.09*	0.84 ± *0.09*	0.91 ± *0.12*	0.90 ± *0.12*	1.02 ± *0.11*	0.95 ± *0.13*	0.90 ± *0.12*	0.96 ± *0.13*
	% further increased	6	2	0	3	34	10	6	14
	% antagonistic	16	75	37	47	34	0	50	23
	P-value*	.19	<.001	.76	<.001				
	P-value**	<.001 (all different)				<.001 (Nuuk different)			

**XAR **RLU/μg protein	*n*	34	33	38	110	41	28	33	102
	median	1.21	1.12	1.13	1.16	1.14	0.99	1.01	1.06
	mean ± SD	1.22 ± *0.27*	1.20 ± *0.29*	1.16 ± *0.27*	1.20 ± *0.27*	1.17 ± *0.29*	1.01 ± *0.18*	1.09 ± *0.27*	1.10 ± *0.26*
	% agonistic	18	22	11	17	15	0	12	10
	% decreased	6	3	0	3	2	0	0	1
	P-value*	.50	.01	.22	.01				
	P-value**	.47 (no post hoc test)				.07 (no post hoc test)			

**XARcomp **RLU/μg protein	*n*	34	33	38	110	41	28	33	102
	median	1.38	1.17	0.84	1.17	0.94	0.80	0.84	0.87
	mean ± SD	1.40 ± *0.27*	1.24 ± *0.37*	0.90 ± *0.22*	1.18 ± *0.36*	0.99 ± *0.22*	0.81 ± *0.13*	0.86 ± *0.21*	0.90 ± *0.21*
	% further increased	50	17	5	23	5	0	6	4
	% antagonistic	0	6	24	11	12	25	21	19
	P-value*	<.001	<.001	.49	<.001				
	P-value**	<.001 (all different)				.001 (Nuuk different)			

**XAR/XER**	*n*	28	33	33	99	41	28	29	98
	mean ± SD	1.19 ± *0.27*	1.29 ± *0.34*	1.19 ± *0.28*	1.23 ± *0.30*	1.14 ± *0.27*	1.09 ± *0.24*	1.08 ± *0.25*	1.11 ± *0.25*
	P-value*	.51	.02	.09	.004				
	P-value**	.46 (no post hoc test)				.64 (no post hoc test)			

**XARcomp/XERcomp**	*n*	28	33	34	100	41	28	29	98
	mean ± SD	1.41 ± *0.29*	1.50 ± *0.47*	1.03 ± *0.31*	1.33 ± *0.43*	0.98 ± *0.22*	0.89 ± *0.20*	0.96 ± *0.26*	0.95 ± *0.23*
	P-value*	<.001	<.001	.36	<.001				
	P-value**	<.001 (Qaanaaq different)				.30 (no post hoc test)			

XER and XAR: ER and AR transactivity of serum extract alone, respectively; XERcomp and XARcomp: competitive receptor transactivity upon co-treatment with EC_50 _of E2 or R1881, respectively.

% agonistic and % decreased indicates a significant increase or decrease, respectively, for the XER and XAR activity compared to the activity of the solvent control which was set to 1. % further increased and % antagonist indicates a further significant increase or decrease, respectively, for the XERcomp and XARcomp transactivity compared to the transactivity of the E2-EC_50_or R1881-EC_50 _solvent control, which was set to 1.

RLU/μg protein: The determined luciferase activity was corrected for cell density using protein measurements and expressed in relative luciferase units per microgram protein (RLU/μg protein).

*: p value for men compared to women within the same district (e.g. Nuuk men and Nuuk women) using Student's t-test.

**: p value for comparison between the districts for the separate genders (e.g. Nuuk vs Sisimiut vs Qaanaaq and vice versa) using ANOVA, result in the brackets (post hoc LSD/Dunnett T3 test).

**Figure 1:**
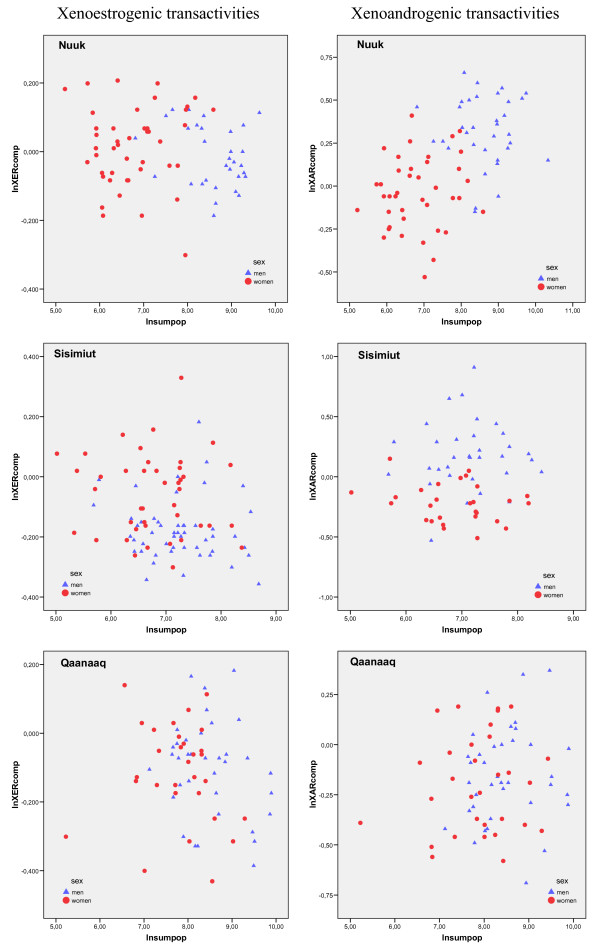
**Xenohormone serum transactivities in the Greenlandic districts**. Scatterplots of the ln-transformed xenoestrogenic and xenoandrogenic serum transactivities versus ΣPOP for men and women in Nuuk, Sisimiut and Qaanaaq.

Scatter plots of the xenoestrogenic transactivities versus ΣPOP for the single districts are shown in figure 1.

#### 3.3.1 Correlation between xenoestrogenic transactivities and lifestyle characteristics

For the combined men positive correlations were observed for XER to n-3/n-6 fatty acid ratio and smoker years and for XERcomp to age and smoker years (Table 4).

**Table 4:** Pearson 2-tailed correlation between xenohormone transactivities and lifestyle characteristics

**Xenoestrogenic transactivities**													
**Male serum**		Nuuk			Sisimiut			Qaanaaq			All		
				
		n	β	p	n	β	p	n	β	p	n	β	p

*XER*	age	32	.19	.30	**51**	**-.45**	**.001**	36	.18	.28	119	.14	.14
	n-3/n-6	32	.29	.11	37	-.25	.14	36	.08	.65	**105**	**.20**	**.04**
	BMI	30	.04	.85	51	-.19	.18	36	-.09	.61	117	-.07	.47
	smokeyrs	29	.28	.15	51	-.26	.07	**35**	**.37**	**.03**	**115**	**.25**	**.01**
	seabird	29	-.25	.20	47	.17	.26	33	-.15	.41	109	.06	.54
	dairy food	31	.06	.74	50	-.08	.59	36	.03	.88	117	-.07	.44

*XERcomp*	age	32	-.11	.56	51	-.05	.71	38	.12	.46	**121**	**.36**	**<.001**
	n-3/n-6	**32**	**-.43**	**.01**	37	-.24	.15	38	-.18	.29	107	.09	.37
	BMI	30	-.29	.12	51	-.08	.58	38	-.17	.31	119	-.09	.31
	smokeyrs	29	.08	.70	51	.20	.16	37	.24	.16	**117**	**.39**	**<.001**
	seabird	29	-.25	.19	47	-.04	.79	35	-.01	.94	111	.12	.21
	dairy food	31	-.09	.64	50	.02	.90	38	-.002	.99	119	-.14	.13

**Female serum**		Nuuk			Sisimiut			Qaanaaq			All		
				
		n	β	p	n	β	p	n	β	p	n	β	p

*XER*	age	**45**	**-.36**	**.02**	42	-.11	.51	32	.23	.21	119	-.01	.93
	n-3/n-6	45	.09	.55	38	-.17	.32	32	.01	.96	115	-.05	.57
	BMI	43	-.17	.27	42	.11	.50	32	-.21	.24	117	-.04	.70
	smokeyrs	**42**	**-.35**	**.02**	42	.05	.77	30	.28	.14	114	.04	.69
	seabird	38	-.04	.81	39	.04	.81	31	.04	.83	108	.05	.63
	dairy food	45	.03	.84	41	-.05	.77	31	-.24	.19	117	-.06	.51

*XERcomp*	age	45	.98	.58	42	-.07	.68	32	-.06	.74	119	.07	.46
	n-3/n-6	45	-.11	.47	38	-.09	.59	32	-.32	.08	**115**	**-.27**	**<.01**
	BMI	43	.04	.80	42	-.05	.77	32	-.18	.32	117	-.05	.61
	smokeyrs	42	.11	.49	42	.10	.53	30	-.06	.74	114	.08	.40
	seabird	**38**	**.34**	**.04**	39	.01	.96	31	-.08	.68	108	-.04	.67
	dairy food	45	-.10	.52	41	.02	.93	31	-.08	.65	117	.05	.59

**Xenoandrogenic transactivities**													

**Male serum**		Nuuk			Sisimiut			Qaanaaq			All		
				
		n	β	p	n	β	p	n	β	p	n	β	p

*XAR*	age	34	-.11	.53	33	.05	.79	38	-.03	.87	105	.02	.82
	n-3/n-6	34	.01	.96	27	-.12	.57	38	.04	.81	99	.001	.99
	BMI	32	-.03	.89	33	-.21	.25	38	-.09	.59	103	-.10	.30
	smokeyrs	31	-.24	.19	33	.12	.49	36	.09	.59	100	-.01	.96
	seabird	31	.19	.32	**32**	**-.35**	**.05**	36	.13	.45	99	.02	.84
	dairy food	33	.20	.25	32	-.21	.26	38	.14	.39	103	.09	.37

*XARcomp*	age	34	-.21	.24	33	.12	.53	38	.09	.61	**105**	**.33**	**.001**
	n-3/n-6	34	.03	.87	27	-.15	.44	38	.003	.99	99	.03	.78
	BMI	32	.07	.70	33	-.17	.33	38	.11	.51	103	.01	.92
	smokeyrs	31	-.32	.08	33	-.04	.81	36	.09	.59	100	.16	.12
	seabird	31	.002	.99	32	-.12	.53	36	.08	.66	99	.13	.19
	dairy food	33	.20	.27	32	-.17	.36	38	.21	.22	103	.17	.09

**Female serum**		Nuuk			Sisimiut			Qaanaaq			All		
				
		n	β	p	n	β	p	n	β	p	n	β	p

*XAR*	age	41	-.11	.49	28	.04	.83	33	-.10	.59	102	-.02	.83
	n-3/n-6	**41**	**.34**	**.03**	26	.08	.69	33	-.19	.28	100	.03	.80
	BMI	39	-.19	.26	28	-.23	.24	33	-.04	.81	100	-.16	.12
	smokeyrs	38	-.25	.13	28	.22	.26	31	-.03	.86	97	-.03	.76
	seabird	36	-.13	.45	27	-.30	.13	32	-.13	.48	95	-.14	.19
	dairy food	41	.08	.60	**27**	**.44**	**.02**	31	-.30	.10	99	.05	.63

*XARcomp*	age	41	-.07	.69	28	-.09	.65	33	-.03	.85	102	.03	.80
	n-3/n-6	41	.19	.22	26	.04	.84	33	.08	.66	100	.002	.98
	BMI	39	.26	.11	28	.20	.31	33	-.04	.84	100	.10	.31
	smokeyrs	38	.01	.97	28	-.09	.65	31	.08	.69	97	.05	.63
	seabird	36	-.11	.52	27	-.07	.72	32	-.15	.43	95	-.14	.18
	dairy food	41	-.04	.81	27	-.04	.86	31	.21	.27	99	.14	.18

For the combined women negative correlations were observed for XERcomp to n-3/n-6 fatty acid ratio (Table 4).

For the single districts correlations between xenoestrogenic transactivities and age, n-3/n-6, seabird and smoker years were observed.

#### 3.3.2 Association between xenoestrogenic transactivities and POPs

Few and scattered significant correlations were observed between the xenoestrogenic transactivities and the single PCBs and pesticides (data not shown).

For the XER data of the combined men and the combined women no significant correlations to the sums of POPs were observed (data not shown). In the single districts, the non-adjusted XER data of Nuuk men correlated positively to ΣPCB (n = 32, β = 0.36, p = 0.04) and ΣPOP (n = 32, β = 0.36, p = 0.04), whereas the XER data of Sisimiut men correlated negatively to ΣPCB (n = 51, β = -0.40, p = 0.004), Σpesticide (n = 51, β = - 0.45, p = 0.001) and ΣPOP (n = 51, β = - 0.43, p = 0.001). No XER-POP correlations were observed for the women in the single districts.

For the XERcomp data of the combined men significant positive correlations were observed to non-adjusted POPs but the correlations disappeared upon adjustment for age and/or n-3/n-6 fatty acid ratio (Table 5). For the XERcomp data of the combined women negative correlations were observed to non-adjusted as well as age adjusted POPs (Table 5). No XERcomp-POP correlations were observed in the single districts.

**Table 5:** Linear regression analyses of xenohormone transactivities before and upon adjustment for lifestyle characteristics

**Xenoestrogenic transactivities (XERcomp)**													
**Male serum**		**Nuuk**			**Sisimiut**			**Qaanaaq**			**All**		
				
		n	β	p	n	β	p	n	β	p	n	β	p

Σ*PCB*	Non-adjusted	32	-.26	.15	51	-.08	.56	36	-.24	.17	**119**	**.22**	**.02**
	+age	-	-	-	-	-	-	-	-	-	119	-.00	.99
	+n-3/n-6	-	-	-	-	-	-	-	-	-	105	.27	.06
	+age+n-3/n-6	-	-	-	-	-	-	-	-	-	105	.10	.47

Σ*pesticide*	Non-adjusted	32	-.32	.07	51	-.13	.37	36	-.21	.21	119	.17	.07
	+age	-	-	-	-	-	-	-	-	-	119	-.07	.50
	+n-3/n-6	-	-	-	-	-	-	-	-	-	105	.13	.39
	+age+n-3/n-6	-	-	-	-	-	-	-	-	-	105	-.03	.86

Σ*POP*	Non-adjusted	32	-.30	.09	51	-.11	.45	36	-.24	.17	**119**	**.20**	**.03**
	+age	-	-	-	-	-	-	-	-	-	119	-.04	.71
	+n-3/n-6	-	-	-	-	-	-	-	-	-	105	.21	.16
	+age+n-3/n-6	-	-	-	-	-	-	-	-	-	105	.04	.80

**Female serum**		**Nuuk**			**Sisimiut**			**Qaanaaq**			**All**		
				
		n	β	p	n	β	p	n	β	p	n	β	p

Σ*PCB*	Non-adjusted	44	.11	.49	42	-.06	.70	30	-.01	.96	116	-.14	.14
	+age	-	-	-	-	-	-	-	-	-	116	-.18	.07
	+n-3/n-6	-	-	-	-	-	-	-	-	-	112	.05	.62
	+age+n-3/n-6	-	-	-	-	-	-	-	-	-	112	.01	.92

Σ*pesticide*	Non-adjusted	44	.02	.90	42	-.09	.58	30	-.14	.48	**116**	**-.20**	**.03**
	+age	-	-	-	-	-	-	-	-	-	**116**	**-.24**	**.01**
	+n-3/n-6	-	-	-	-	-	-	-	-	-	112	-.02	.84
	+age+n-3/n-6	-	-	-	-	-	-	-	-	-	112	-.06	.58

Σ*POP*	Non-adjusted	44	.05	.74	42	-.08	.62	30	-.10	.61	116	-.18	.06
	+age	-	-	-	-	-	-	-	-	-	**116**	**-.22**	**.03**
	+n-3/n-6	-	-	-	-	-	-	-	-	-	112	.01	.94
	+age+n-3/n-6	-	-	-	-	-	-	-	-	-	112	-.04	.76

**Xenoandrogenic transactivities(XARcomp)**													

**Male serum**		**Nuuk**			**Sisimiut**			**Qaanaaq**			**All**		
				
		n	β	p	n	β	p	n	β	p	n	β	p

Σ*PCB*	Non-adjusted	34	-.07	.70	33	.003	.99	37	.17	.32	104	-.05	.62*
	+age	-	-	-	-	-	-	-	-	-	**104**	**-.39**	**.001**
	+n-3/n-6	-	-	-	-	-	-	-	-	-	98	-.22	.14
	+age+n-3/n-6	-	-	-	-	-	-	-	-	-	**98**	**-.42**	**<.01**

Σ*pesticide*	Non-adjusted	34	-.01	.96	33	.06	.76	37	.10	.54	104	-.04	68*
	+age	-	-	-	-	-	-	-	-	-	**104**	**-.36**	**<.01**
	+n-3/n-6	-	-	-	-	-	-	-	-	-	98	-.20	.19
	+age+n-3/n-6	-	-	-	-	-	-	-	-	-	**98**	**-.36**	**.01**

Σ*POP*	Non-adjusted	34	-.04	.83	33	.04	.85	37	.13	.44	104	-.04	.67*
	+age	-	-	-	-	-	-	-	-	-	**104**	**-.38**	**.001**
	+n-3/n-6	-	-	-	-	-	-	-	-	-	98	-.21	.17
	+age+n-3/n-6	-	-	-	-	-	-	-	-	-	**98**	**-.40**	**<.01**

**Female serum**		**Nuuk**			**Sisimiut**			**Qaanaaq**			**All**		
				
		n	β	p	n	β	p	n	β	p	n	β	p

Σ*PCB*	Non-adjusted	40	.09	.60	28	-.21	.29	31	.13	.47	99	-.06	.56
	+age	-	-	-	-	-	-	-	-	-	99	-.07	.50
	+n-3/n-6	-	-	-	-	-	-	-	-	-	97	-.04	.75
	+age+n-3/n-6	-	-	-	-	-	-	-	-	-	97	-.06	.68

Σ*pesticide*	Non-adjusted	40	.15	.35	28	-.26	.19	31	.08	.67	99	-.06	55
	+age	-	-	-	-	-	-	-	-	-	99	-.07	.50
	+n-3/n-6	-	-	-	-	-	-	-	-	-	97	-.05	.74
	+age+n-3/n-6	-	-	-	-	-	-	-	-	-	97	-.06	.67

Σ*POP*	Non-adjusted	40	.13	.43	28	-.23	.24	31	.10	.59	99	-.06	57
	+age	-	-	-	-	-	-	-	-	-	99	-.07	.52
	+n-3/n-6	-	-	-	-	-	-	-	-	-	97	-.04	.77
	+age+n-3/n-6	-	-	-	-	-	-	-	-	-	97	-.06	.70

Adjustment for BMI, smoker years and bird intake did not further change the data.

The overall association between the POP markers and xenohormone transactivity across the study groups were evaluated using multiple linear regressions analysis. Homogeneity across the districts was observed (see Additional file 1) and to improve the statistical power and the overall trend of the study groups the combined male and female data across the districts was evaluated.

*: The combined data of Sisimiut and Qaanaaq men was significantly negatively correlated to non-adjusted as well as age adjusted POPs.

-: The data of the single districts were not adjusted for lifestyle characteristics because of the few cases in each district (n = 31 – 40).

### 3.4 Xenoandrogenic transactivities

Table 3 shows that in overall the serum POP extracts agonized the XAR transactivities while antagonized XARcomp transactivities were observed except for Nuuk and Sisimiut men. The XAR transactivities for Sisimiut men were significantly higher than for Sisimiut women and the XARcomp transactivities for Nuuk and Sisimiut men were significantly higher than for Nuuk and Sisimiut women, respectively. Furthermore, the ANOVA analysis showed significant differences between districts for the XARcomp transactivities of both genders (Table 3).

Scatter plots of the xenoandrogenic transactivities and ΣPOP for the single districts are shown in Figure 1.

#### 3.4.1 Correlation between xenoandrogenic transactivities and lifestyle characteristics

The XAR transactivities did not correlated to any lifestyle characteristics for the combined men or the combined women, whereas the XARcomp transactivities correlated positively to age for the combined men. Few and scattered correlations were found for the single districts (Table 4).

#### 3.4.2 Association between xenoandrogenic transactivities and POPs

Few and scattered significant correlations were observed between the xenoandrogenic transactivities and the single PCBs and pesticides (data not shown). For the XAR transactivity data no correlations were observed to the sums of POPs neither for the combined men, the combined women nor in the single districts (data not shown).

The combined male data across the districts showed negative correlations between XARcomp and the POPs upon adjustment for age and age plus n-3/n-6 ratio. Excluding the Nuuk men, being atypically with respect to age and diet (see section 3.1) compared to Sisimiut and Qaanaaq men, a significant negative correlation between XARcomp and the POPs were observed both before and after adjustment for age. No XARcomp-POP correlations were observed for the combined women, neither in the single districts (Table 5).

### 3.5 The ratio of xenoandrogenic to xenoestrogenic serum transactivity

The XAR/XER ratio suggests a net xenoandrogenicity (ratio > 1) for both genders and also the XARcomp/XERcomp ratio for men. However, for the women the XARcomp/XERcomp ratio being < 1 suggests a net xenoestrogenic activity (Table 3).

## 4. Discussion

In the present study we determined the actual xenohormone transactivity as a biomarker of serum POP exposure and evaluated whether the transactivity was associated to serum POP concentrations and the lifestyle characteristics across the Greenlandic districts Nuuk, Sisimiut and Qaanaaq. For the age and/or n-3/n-6 adjusted data we found an inverse correlation between xenoestrogenic and xenoandrogenic transactivities for combined female and combined male, respectively.

In general, serum POP extracts, free of endogenous hormones, showed a trend to decrease the ER transactivity (XER) and a low trend to agonize the AR transactivity (XAR) for both genders. In order to assure that the effect of the actual serum POP mixture on the receptor activation does reflect the biological activity in serum and the *in vivo *interaction between endogenous hormones and the POPs, we did evaluate the effect of the serum POP extracts in the presence of relevant concentrations of high affinity ligands (E2 and R1881) for the receptors to mimic the *in vivo *potential of the serum samples (XERcomp and XARcomp).

In the single districts XERcomp and XARcomp transactivities were predominantly antagonized except for serum extracts of the older Nuuk men where 50% elicited further increased XARcomp transactivities. No significant differences in the XER and the XAR were observed between men and women except the XAR activity of Sisimiut men being higher than Sisimiut women supporting that the endogenous hormones were effectively separated from the serum POP fractions.

Because of differences between the genders in lifestyle characteristics and POP levels and to improve the statistical power, we evaluated the combined data of each gender across the districts.

In the present study the serum samples of men from Nuuk contained a higher level of POPs due to their higher average age and higher intake of seafood and seabird [7]. However, in accordance with previous studies, Qaanaaq inhabitants, having a high intake of marine and traditional diet, had in general significantly higher levels of POPs compared to Nuuk women and Sisimiut inhabitants, eating a more westernized diet. For all three districts the serum POP levels of men were significantly higher than for women, reflecting the higher intake of traditional food by men [7]. Moreover, despite a high intercorrelation between POPs, the profile differed between districts and genders reflecting different exposure pattern.

In Arctic populations higher serum POP concentrations is determined by age and intake of marine food as reflected by an increased n-3/n-6 fatty acid ratio [8,42], and a higher level of POPs was reported for smokers [7,9]. We observed positive associations between the xenohormone transactivities and age, n-3/n-6 and smoker years for the combined male data whereas the xenoestrogenic activities were negatively associated to n-3/n-6 for the combined women. Thus, the determinants of the serum POP concentration seem also to be predictors of the xenohormone transactivities supporting the use of xenohormone transactivities as an integrated biomarker of POP exposure and lifestyle characteristics.

For combined men the positive correlation between the XERcomp transactivities and the POPs disappeared upon adjustment for age. POP concentrations increase by age, and age was found to be a determinant of the XERcomp transactivities for men. Thus, the observed association between XERcomp and the unadjusted POP data might be a reflection of the broad range of age for the included men and the correlation between XERcomp and age.

The XERcomp transactivities for the combined women correlated inversely to both non-adjusted and age-adjusted POPs. We found in a previous study [33], evaluating the xenoestrogenic serum transactivity of male study groups from Europe and Greenland, a negative correlation between the ER transactivity and the two POP proxy markers PCB153 and *p,p'*-DDE for Inuit.

Dioxins are known to exert antiestrogenic actions [43,44]. In the same study population as used in this study, the serum dioxin-like transactivity mediated via the aryl hydrocarbon receptor (AhR) was determined [45]. Interestingly, the xenoestrogenic transactivities correlated negatively to the AhR transactivities for the combined women supporting the presence of antiestrogenic POPs in the serum POP mixture. In support, although using another approach it was recently reported that high levels of PCBs in Slovakia male serum samples were associated with a decreased ER mediated activity and increased AhR mediated activity [41].

Data on the xenoestrogenic activity in human samples are still scarce. Few studies using a different setup of measurement of xenoestrogenic transactivities in adipose tissue from Spanish women and serum samples from pregnant Danish and Faroese women have been reported, although, in contrast to our studies no correlation between POPs and MCF-7 proliferation were found [32,20,31,34]. However, recent data showed, for non-pregnant female greenhouse workers in Denmark that the E2-induced MCF-7 proliferation response was reduced, indicating an antiestrogenic effect of the serum extract containing major xenoestrogens but without pharmaceutical and endogenously produced estrogens [46]. We believe that the high impact of POP burden in the Inuit is responsible for the antagonized ER function in line with earlier *in vitro* studies [15,34].

In the present study only a small fraction of the serum POP extracts agonized the XAR transactivities. Numerous POPs have been shown to be antiandrogenic *in vivo *and *in vitro *[17,47,14,18,15-51]. However, also androgenic effects have been found in the environment, e.g. craft mill effluent from a river in Florida contained a chemical mixture that induced AR-dependent gene expression and masculinized female fish *in vitro *[52].

The *in vivo *mimicking XARcomp transactivities were predominantly antagonized supporting the *in vivo *and *in vitro *antiandrogenic effects of the POPs. However, for 50% of the older Nuuk men a further increased XARcomp transactivity was observed. Some AhR agonists elicit antiandrogenic effects although the exact mechanisms are not clear [53-55]. In the same study population as used in this study, the 2,3,7,8-tetrachlorodibenzo-*p*-dioxin (TCDD)-induced AhR transactivities (AhRcomp) were significantly lower for Nuuk men compared to men from Sisimiut and Qaanaaq [45]. Furthermore, 32% of the serum samples of Nuuk men inhibited AhRcomp transactivity compared to 4% and 2% of the serum samples of Sisimiut and Qaanaaq men, respectively [45]. Thus, the serum extracts of Nuuk men seems to include some POPs further increasing the XARcomp transactivity but inhibiting the AhRcomp transactivity. Whether these POPs are related to the higher age of Nuuk men or their higher intake of seafood and seabird can at this point not be determined.

Taking the broad age range of the men across the districts into account the combined male XARcomp data correlated negatively to the POPs upon adjustment for age. Because of the higher age of Nuuk men we also analyzed the combined data of Sisimiut and Qaanaaq men separately, and significant negative correlations to the POPs were observed both before and after adjustment for age. In support to the present results, we previously observed negative correlations between XARcomp and the POP proxy marker *p,p'*-DDE for combined European study groups [36].

The data showed correlations between POPs and female xenoestrogenic and male xenoandrogenic transactivities. We cannot explain this gender pattern but have the following suggestions: The serum POP concentration was approximately 3 times higher in male serum compared to female serum mainly because of a different diet pattern, men eating more traditional and women more westernized [7], resulting in a different compositions of the serum POP mixture between the two sexes.

We believe that the presented data reflects the effect of POPs on the sex hormone receptors as supported by the following: the observed correlations between xenohormone transactivities and the POPs were all observed in the *in vivo *mimic by the presence of a high affinity receptor ligand (E2 for ER and R1881 for AR). No correlations were observed for the serum POP extracts alone and furthermore, the observed correlations were negative. Thus, it does not seem plausible that the correlations between XERcomp/XARcomp and the age-adjusted POPs result from remnant endogenous hormones in the serum POP fraction. However, we cannot assess whether the correlations in the present study are only by chance findings. 25 out of 276 correlations (<10%) showed statistically significance, whereas the expected number of significant findings under the null hypothesis of no difference would be 14 out of 276 correlations (5%). We did observe fewer significant findings for the xenoandrogenic transactivities. This could be a result of the similar coefficients of variation within and between days for the AR-CALUX assay making the assay less robust than the ER assay.

Interestingly, we found higher xenoandrogenic/xenoestrogenic ratio for the male Inuit compared to the daily serum controls of Danish men (see section 2.3) as well as compared to European male in another study [36]. At this point it is not possible to assess whether this change in xenoandrogenicity can have any impact *in vivo *on the hormone homeostasis. It was proposed, that exposure to a mixture of chemicals with antiandrogenic or estrogenic properties may affect the androgen-estrogen ratio and thereby influence the risk of cryptorchidism in Danish and Finnish boys [56]. Whether a lower rate of cryptorchidism is found in Inuit awaits further research. We did previously observe negative correlations for xenoestrogenic and dioxin-like transactivity to sperm chromatin integrity and DNA damage for Inuit and positive correlations between xenoandrogenic and dioxin-like transactivity and sperm chromatin integrity and DNA damage for Europeans [57,58]. Further studies are needed to elucidate the possible association between POP related xenohormone transactivity and human health risk. Furthermore, other types of lipophilic endocrine disrupters in serum like the brominated flame retardants (BFRs) and perfluorooctanesulfonated (PFOS) affect the receptor function and should in future studies be taken into consideration.

Whether our *ex vivo *data mimic the cellular and molecular effects *in vivo *awaits additional studies. The presented data gives the final integrated net effect on the given receptor alone and other factors such as receptor and/or co-factor interactions might precede the final data. Our biomarker approach gives the possibility to estimate the combined impact of complex mixtures on health risks mediated via sex hormone receptors.

## 5. Conclusion and Perspectives

Determinants of serum POP concentration are also predictors of the xenohormone transactivities and can be used as an integrated biomarker of POP exposure and lifestyle characteristics. The actual serum POP mixture antagonize age adjusted sex hormone actions as supported by the inverse correlations to the xenoestrogenic and the xenoandrogenic transactivities for the combined female and the combined male Inuit, respectively.

The data of the presented manuscript is the first of its kind ever published for Inuit having very high levels of POPs. The emerging changes in diet in Greenland and thus the observed district differences in POP concentrations as well as mixture profile will be of great importance for the future elucidation of how mixtures of different POP levels and mixtures among different geographical population might affect the risk of health.

## Competing interests

The authors declare that they have no competing interests.

## Authors' contributions

ECBJ was responsible for the design of the study. TK, MG and ECBJ drafted the manuscript and were responsible for data evaluation and statistical analyses. TK carried out the AR-CALUX analyses. MG carried out the ER-CALUX analyses. PSH carried out the SPE-HPLC fractionation of the serum samples and commented on the manuscript. BD evaluated the POP data and questionnaires concerning lifestyle characteristics and established the database. All authors read and approved the final manuscript.

## Supplementary Material

Additional file 1 Multiple regressions of the combined study groups. The data provided represent the multiple linear regressions analysis of homogeneity or heterogeneity between the POP markers and xenohormone transactivity for each gender across the study groups. Click here for file

## References

[B1] Barrie LA, Gregor D, Hargrave B, Lake R, Muir D, Shearer R, Tracey B, Bidleman T (1992). Arctic contaminants: sources, occurrence and pathways. Sci Total Environ.

[B2] Macdonal RW, Barrie LA, Bidleman TF, Diamond ML, Gregor DJ, Semkin RG, Strachan WM, Li YF, Wania F, Alaee M, Alexeeva LB, Backus SM, Bailey R, Bewers JM, Gobeil C, Halsall CJ, Harner T, Hoff JT, Jantunen LM, Lockhart WL, Mackay D, Muir DC, Pudykiewicz J, Reimer KJ, Smith JN, Stern GA (2000). Contaminants in the Canadian Arctic: 5 years of progress in understanding sources, occurrence and pathways. Sci Total Environ.

[B3] Dewailly E, Ayotte P, Bruneau S, Laliberte C, Muir DC, Norstrom RJ (1993). Inuit exposure to organochlorines through the aquatic food chain in arctic quebec. Environ Health Perspect.

[B4] Van Oostdam JC, Dewailly E, Gilman A, Hansen JC, Odland JO, Chashchin V, Berner J, Butler-Walker J, Lagerkvist BJ, Olafsdottir K, Soininen L, Bjerregard P, Klopov V, Weber JP (2004). Circumpolar maternal blood contaminant survey, 1994-1997 organochlorine compounds. Sci Total Environ.

[B5] Cote S, Dodin S, Blanchet C, Mulvad G, H.S.. P, Blanchet C, Holub BJ, Dewailly E (2004). Very high concentrations of n-3 fatty acids in peri- and postmenopausal Inuit women from Greenland. Int J Circumpolar Health.

[B6] Deutch B, Dyerberg J, Pedersen HS, Asmund G, Moller P, Hansen JC (2006). Dietary composition and contaminants in north Greenland, in the 1970s and 2004. Sci Total Environ.

[B7] Deutch B, Pedersen HS, Asmund G, Hansen JC (2007). Contaminants, diet, plasma fatty acids and smoking in Greenland 1999-2005. Sci Total Environ.

[B8] Deutch B, Pedersen HS, Hansen JC (2004). Dietary composition in Greenland 2000, plasma fatty acids and persistent organic pollutants. Sci Total Environ.

[B9] Deutch B, Pedersen HS, Jorgensen EC, Hansen JC (2003). Smoking as a determinant of high organochlorine levels in Greenland. Arch Environ Health.

[B10] Deutch B, Hansen JC (2000). High human plasma levels of organochlorine compounds in Greenland. Regional differences and lifestyle effects. Dan Med Bull.

[B11] Riget F, Dietz R, Vorkamp K, Johansen P, Muir D (2004). Levels and spatial and temporal trends of contaminants in Greenland biota: an updated review. Sci Total Environ.

[B12] Vorkamp K, Christensen JH, Glasius M, Riget FF (2004). Persistent halogenated compounds in black guillemots (Cepphus grylle) from Greenland--levels, compound patterns and spatial trends. Mar Pollut Bull.

[B13] Dewailly E, Mulvad G, Pedersen HS, Ayotte P, Demers A, Weber JP, Hansen JC (1999). Concentration of organochlorines in human brain, liver, and adipose tissue autopsy samples from Greenland. Environ Health Perspect.

[B14] Andersen HR, Vinggaard AM, Rasmussen TH, Gjermandsen IM, Bonefeld-Jorgensen EC (2002). Effects of currently used pesticides in assays for estrogenicity, androgenicity, and aromatase activity in vitro. Toxicol Appl Pharmacol.

[B15] Bonefeld-Jorgensen EC, Andersen HR, Rasmussen TH, Vinggaard AM (2001). Effect of highly bioaccumulated polychlorinated biphenyl congeners on estrogen and androgen receptor activity. Toxicology.

[B16] Bonefeld-Jorgensen EC, Autrup H, Hansen JC (1997). Effect of toxaphene on estrogen receptor functions in human breast cancer cells. Carcinogenesis.

[B17] Kelce WR, Stone CR, Laws SC, Gray LE, Kemppainen JA, Wilson EM (1995). Persistent DDT metabolite p,p'-DDE is a potent androgen receptor antagonist. Nature.

[B18] Roy P, Salminen H, Koskimies P, Simola J, Smeds A, Saukko P, Huhtaniemi IT (2004). Screening of some anti-androgenic endocrine disruptors using a recombinant cell-based in vitro bioassay. J Steroid Biochem Mol Biol.

[B19] Bonefeld-Jørgensen EC, Ayotte P (2003). Toxicological Properties of POPs and Related Health Effects of Concern for the Arctic Populations.. AMAP assessment 2002: Human Health in the Arctic.

[B20] Ibarluzea JM, Fernandez MF, Santa-Marina L, Olea-Serrano MF, Rivas AM, Aurrekoetxea JJ, Exposito J, Lorenzo M, Torne P, Villalobos M, Pedraza V, Sasco AJ, Olea N (2004). Breast cancer risk and the combined effect of environmental estrogens. Cancer Causes & Control.

[B21] Ahmed SA (2000). The immune system as a potential target for environmental estrogens (endocrine disrupters): a new emerging field. Toxicology.

[B22] Hotchkiss AK, Ostby JS, Vandenburgh JG, Gray LE (2002). Androgens and environmental antiandrogens affect reproductive development and play behavior in the Sprague-Dawley rat. Environ Health Perspect.

[B23] McLachlan JA, Newbold RR, Burow ME, Li SF (2001). From malformations to molecular mechanisms in the male: three decades of research on endocrine disrupters. Apmis.

[B24] Patandin S, Lanting CI, Mulder PG, Boersma ER, Sauer PJ, Weisglas-Kuperus N (1999). Effects of environmental exposure to polychlorinated biphenyls and dioxins on cognitive abilities in Dutch children at 42 months of age. J Pediatr.

[B25] Guillette EA, Conard C, Lares F, Aguilar MG, McLachlan J, Guillette LJ (2006). Altered breast development in young girls from an agricultural environment. Environ Health Perspect.

[B26] Main KM, Mortensen GK, Kaleva MM, Boisen KA, Damgaard IN, Chellakooty M, Schmidt IM, Suomi AM, Virtanen HE, Petersen DV, Andersson AM, Toppari J, Skakkebaek NE (2006). Human breast milk contamination with phthalates and alterations of endogenous reproductive hormones in infants three months of age. Environ Health Perspect.

[B27] Swan SH, Main KM, Liu F, Stewart SL, Kruse RL, Calafat AM, Mao CS, Redmon JB, Ternand CL, Sullivan S, Teague JL (2005). Decrease in anogenital distance among male infants with prenatal phthalate exposure. Environ Health Perspect.

[B28] Payne J, Scholze M, Kortenkamp A (2001). Mixtures of four organochlorines enhance human breast cancer cell proliferation. Environ Health Perspect.

[B29] Rajapakse N, Silva E, Kortenkamp A (2002). Combining xenoestrogens at levels below individual no-observed-effect concentrations dramatically enhances steroid hormone action. Environ Health Perspect.

[B30] Metzdorff SB, Dalgaard M, Christiansen S, Axelstad M, Hass U, Kiersgaard MK, Scholze M, Kortenkamp A, Vinggaard AM (2007). Dysgenesis and histological changes of genitals and perturbations of gene expression in male rats after in utero exposure to antiandrogen mixtures. Toxicol Sci.

[B31] Rivas A, Fernandez MF, Cerrillo I, Ibarluzea J, Olea-Serrano MF, Pedraza V, Olea N (2001). Human exposure to endocrine disrupters: standardisation of a marker of estrogenic exposure in adipose tissue. Apmis.

[B32] Fernandez MF, Rivas A, Olea-Serrano F, Cerrillo I, Molina-Molina JM, Araque P, Martinez-Vidal JL, Olea N (2004). Assessment of total effective xenoestrogen burden in adipose tissue and identification of chemicals responsible for the combined estrogenic effect. Anal Bioanal Chem.

[B33] Bonefeld-Jorgensen EC, Hjelmborg PS, Reinert TS, Andersen BS, Lesovoy V, Lindh CH, Hagmar L, Giwercman A, Erlandsen M, Manicardi GC, Spano M, Toft G, Bonde JP (2006). Xenoestrogenic activity in blood of European and Inuit populations. Environ Health.

[B34] Rasmussen TH, Nielsen F, Andersen HR, Nielsen JB, Weihe P, Grandjean P (2003). Assessment of xenoestrogenic exposure by a biomarker approach: application of the E-Screen bioassay to determine estrogenic response of serum extracts. Environ Health.

[B35] Hjelmborg PS, Ghisari M, Bonefeld-Jorgensen EC (2006). SPE-HPLC purification of endocrine-disrupting compounds from human serum for assessment of xenoestrogenic activity. Anal Bioanal Chem.

[B36] Krüger T, Hjelmborg PS, Jönsson BAG, Hagmar L, Giwercman A, Manicardi GC, Bizzaro D, Spanò M, Rignell-Hydbom A, Pedersen HS, Toft G, Bonde JP, Bonefeld-Jorgensen EC (2007). Xeno-androgenic activity in serum differs across European and Inuit populations. Environmental Health Perspectives.

[B37] Long M, Andersen BS, Lindh CH, Hagmar L, Giwercman A, Manicardi GC, Bizzaro D, Spano M, Toft G, Pedersen HS, Zvyezday V, Bonde JP, Bonefeld-Jorgensen EC (2006). Dioxin-like activities in blood across European and Inuit populations. Environ Health.

[B38] Butler Walker J, Seddon L, McMullen E, Houseman J, Tofflemire K, Corriveau A, Weber JP, Mills C, Smith S, Van Oostdam J (2003). Organochlorine levels in maternal and umbilical cord blood plasma in Arctic Canada. Sci Total Environ.

[B39] Bonefeld-Jorgensen EC, Grunfeld HT, Gjermandsen IM (2005). Effect of pesticides on estrogen receptor transactivation in vitro: a comparison of stable transfected MVLN and transient transfected MCF-7 cells. Mol Cell Endocrinol.

[B40] Jonsson BA, Rylander L, Lindh C, Rignell-Hydbom A, Giwercman A, Toft G, Pedersen HS, Ludwicki JK, Goralczyk K, Zvyezday V, Spano M, Bizzaro D, Bonefeld-Jorgensen EC, Manicardi GC, Bonde JP, Hagmar L (2005). Inter-population variations in concentrations, determinants of and correlations between 2,2',4,4',5,5'-hexachlorobiphenyl (CB-153) and 1,1-dichloro-2,2-bis (p-chlorophenyl)-ethylene (p,p'-DDE): a cross-sectional study of 3161 men and women from Inuit and European populations. Environ Health.

[B41] Pliskova M, Vondracek J, Canton RF, Nera J, Kocan A, Petrik J, Trnovec T, Sanderson T, van den Berg M, Machala M (2005). Impact of polychlorinated biphenyls contamination on estrogenic activity in human male serum. Environ Health Perspect.

[B42] Tjonneland A, Overvad K, Thorling E, Ewertz M (1993). Adipose tissue fatty acids as biomarkers of dietary exposure in Danish men and women. Am J Clin Nutr.

[B43] Safe S, Wang F, Porter W, Duan R, McDougal A (1998). Ah receptor agonists as endocrine disruptors: antiestrogenic activity and mechanisms. Toxicol Lett.

[B44] Safe S, Wormke M (2003). Inhibitory aryl hydrocarbon receptor-estrogen receptor alpha cross-talk and mechanisms of action. Chem Res Toxicol.

[B45] Long M, Deutch B, Bonefeld-Jorgensen EC (2007). AhR transcriptional activity in serum of Inuits across Greenlandic districts. Environ Health.

[B46] Andersen HR, Nielsen F, Nielsen JB, Kjaerstad MB, Baelum J, Grandjean P (2007). Xeno-oestrogenic activity in serum as marker of occupational pesticide exposure. Occup Environ Med.

[B47] Kelce WR, Monosson E, Gamcsik MP, Laws SC, Gray LE (1994). Environmental hormone disruptors: evidence that vinclozolin developmental toxicity is mediated by antiandrogenic metabolites. Toxicol Appl Pharmacol.

[B48] Birkhoj M, Nellemann C, Jarfelt K, Jacobsen H, Andersen HR, Dalgaard M, Vinggaard AM (2004). The combined antiandrogenic effects of five commonly used pesticides. Toxicol Appl Pharmacol.

[B49] Endo F, Monsees TK, Akaza H, Schill WB, Pflieger-Bruss S (2003). Effects of single non-ortho, mono-ortho, and di-ortho chlorinated biphenyls on cell functions and proliferation of the human prostatic carcinoma cell line, LNCaP. Reprod Toxicol.

[B50] Schrader TJ, Cooke GM (2003). Effects of Aroclors and individual PCB congeners on activation of the human androgen receptor in vitro. Reprod Toxicol.

[B51] Vinggaard AM, Nellemann C, Dalgaard M, Jorgensen EB, Andersen HR (2002). Antiandrogenic effects in vitro and in vivo of the fungicide prochloraz. Toxicol Sci.

[B52] Parks LG, Lambright CS, Orlando EF, Guillette LJ, Ankley GT, Gray LE (2001). Masculinization of female mosquitofish in Kraft mill effluent-contaminated Fenholloway River water is associated with androgen receptor agonist activity. Toxicol Sci.

[B53] Jana NR, Sarkar S, Ishizuka M, Yonemoto J, Tohyama C, Sone H (1999). Cross-talk between 2,3,7,8-tetrachlorodibenzo-p-dioxin and testosterone signal transduction pathways in LNCaP prostate cancer cells. Biochem Biophys Res Commun.

[B54] Kizu R, Okamura K, Toriba A, Kakishima H, Mizokami A, Burnstein KL, Hayakawa K (2003). A role of aryl hydrocarbon receptor in the antiandrogenic effects of polycyclic aromatic hydrocarbons in LNCaP human prostate carcinoma cells. Arch Toxicol.

[B55] Vinggaard AM, Hnida C, Larsen JC (2000). Environmental polycyclic aromatic hydrocarbons affect androgen receptor activation in vitro. Toxicology.

[B56] Toppari J, Virtanen H, Skakkebaek NE, Main KM (2006). Environmental effects on hormonal regulation of testicular descent. J Steroid Biochem Mol Biol.

[B57] Kruger T, Spano M, Long M, Eleuteri P, Rescia M, Hjelmborg PS, Manicardi GC, Bizzaro D, Giwercman A, Toft G, Bonde JP, Bonefeld-Jorgensen EC (2008). Xenobiotic activity in serum and sperm chromatin integrity in European and inuit populations. Mol Reprod Dev.

[B58] Long M, Stronati A, Bizzaro D, Kruger T, Manicardi GC, Hjelmborg PS, Spano M, Giwercman A, Toft G, Bonde JP, Bonefeld-Jorgensen EC (2007). Relation between serum xenobiotic-induced receptor activities and sperm DNA damage and sperm apoptotic markers in European and Inuit populations. Reproduction.

