# Supplementary material for: Xenohormone transactivities are inversely associated to serum POPs in Inuit
Source: Environ Health. 2008 Jul 15;7:38. doi: 10.1186/1476-069X-7-38 (PMC2503956; doi:10.1186/1476-069X-7-38)
Supplement: Additional file 1 — Multiple regressions of the combined study groups. The data provided represent the multiple linear regressions analysis of homogeneity or heterogeneity between the POP markers and xenohormone transactivity for each gender across the study groups. [file 1476-069X-7-38-S1.doc]

**Additional file 1. Multiple regressions** of the combined study groups

| **Across districts for men** | | | | |
| --- | --- | --- | --- | --- |
| Response variable | Homogeneity of slope  (p-value) | Slope  (estimate (SE), p-value) | Intercept  (p-value) | R^2^ |
| **XER (n=117)** | | | | |
| ∑PCB | **0.004** | -* | -* | -* |
| ∑pesticide | **0.001** | -* | -* | -* |
| ∑POP | **0.001** | -* | -* | -* |
| **XERcomp (n=119)** | | | | |
| ∑PCB | 0.58 | -0.03 (0.02), 0.06 | **<0.001** | 0.27 |
| ∑pesticide | 0.79 | **-0.03 (0.01), 0.03** | **<0.001** | 0.27 |
| ∑POP | 0.67 | **-0.03 (0.01), 0.03** | **<0.001** | **0.27** |
| **XAR (n=104)** | | | | |
| ∑PCB | 0.91 | -0.02 (0.04), 0.68 | 0.76 | -0.02** |
| ∑pesticide | 0.99 | -0.01 (0.03), 0.68 | 0.77 | -0.02** |
| ∑POP | 0.98 | -0.01 (0.03), 0.69 | 0.76 | -0.02** |
| **XARcomp (n=104)** | | | | |
| ∑PCB | 0.63 | 0.01 (0.04), 0.71 | **<0.001** | 0.40 |
| ∑pesticide | 0.90 | 0.02 (0.03), 0.59 | **<0.001** | 0.40 |
| ∑POP | 0.80 | 0.02 (0.03), 0.64 | **<0.001** | 0.40 |
| **Across districts for women** | | | | |
| Response variable | Homogeneity of slope  (p-value) | Common slope  (estimate (SE), p-value) | Common intercept  (p-value) | R^2^ |
| **XER (n=116)** | | | | |
| ∑PCB | 0.33 | 0.01 (0.02), 0.57 | **0.001** | 0.10 |
| ∑pesticide | 0.61 | 0.002 (0.01), 0.87 | **0.001** | 0.10 |
| ∑POP | 0.51 | 0.01 (0.02), 0.74 | **0.001** | 0.10 |
| **XERcomp (n=116)** | | | | |
| ∑PCB | 0.77 | 0.002 (0.02), 0.92 | **0.001** | 0.11 |
| ∑pesticide | 0.77 | -0.01 (0.02), 0.47 | **0.002** | 0.12 |
| ∑POP | 0.78 | -0.01 (0.02), 0.67 | **0.002** | 0.11 |
| **XAR (n=99)** | | | | |
| ∑PCB | 0.69 | 0.01 (0.03), 0.78 | 0.07 | 0.03 |
| ∑pesticide | 0.56 | 0.004 (0.03), 0.88 | 0.07 | 0.03 |
| ∑POP | 0.61 | 0.05 (0.03), 0.84 | 0.07 | 0.03 |
| **XARcomp (n=99)** | | | | |
| ∑PCB | 0.48 | 0.01 (0.03), 0.67 | **0.001** | 0.11 |
| ∑pesticide | 0.37 | 0.01 (0.03), 0.69 | **0.001** | 0.11 |
| ∑POP | 0.44 | 0.01 (0.03), 0.66 | **0.001** | 0.11 |

Homogeneity of slope: test for homogeneity of association between exposure variables and outcome variables across the study group (p > 0.05, accept the hypotheses of homogeneity of slope). Slope: the estimated common slope across study groups assuming homogeneity (p > 0.05, accept the hypotheses that slope equals to zero). Intercept: test of a common intercept across study groups assuming a common slope (p > 0.05, accept the hypotheses having common intercept across the study groups). R^2^ (Adjusted R square) assumes a common slope. Bold values are those showing significance.

*: Since heterogeneity of slope exists between POPs and XER men data across the districts, i.e. there were district differences in the associations of POP and XER, no further evaluation was performed.

**: equals to zero
